# Boldine Improves Kidney Damage in the Goldblatt 2K1C Model Avoiding the Increase in TGF-β

**DOI:** 10.3390/ijms19071864

**Published:** 2018-06-25

**Authors:** Gonzalo I. Gómez, Victoria Velarde

**Affiliations:** 1 Departamento de Fisiología, Facultad de Ciencias Biológicas, Pontificia Universidad Católica de Chile, Alameda #340, Santiago 8331150, Chile; 2 Instituto de Ciencias Biomédicas, Facultad de Ciencias de la Salud, Universidad Autónoma de Chile, El Llano Subercaseaux #2801, Santiago 8910060, Chile

**Keywords:** renovascular hypertension, chronic kidney disease, oxidative stress, fibrosis, (S)-2,9-dihydroxy-1,10-dimethoxy-aporphine

## Abstract

Boldine, a major aporphine alkaloid found in the Chilean boldo tree, is a potent antioxidant. Oxidative stress plays a detrimental role in the pathogenesis of kidney damage in renovascular hypertension (RVH). The activation of the renin-angiotensin system (RAS) is crucial to the development and progression of hypertensive renal damage and TGF-β is closely associated with the activation of RAS. In the present study, we assessed the effect of boldine on the progression of kidney disease using the 2K1C hypertension model and identifying mediators in the RAS, such as TGF-β, that could be modulated by this alkaloid. Toward this hypothesis, rats (*n* = 5/group) were treated with boldine (50 mg/kg/day, gavage) for six weeks after 2K1C surgery (pressure ≥ 180 mmHg). Kidney function was evaluated by measuring of proteinuria/creatininuria ratio (U prot/U Crea), oxidative stress (OS) by measuring thiobarbituric acid reactive substances (TBARS). The evolution of systolic blood pressure (SBP) was followed weekly. Alpha-smooth muscle actin (α-SMA) and Col III were used as markers of kidney damage; ED-1 and osteopontin (OPN) were used as markers of inflammation. We also explored the effect in RAS mediators, such as ACE-1 and TGF-β. Boldine treatment reduced the UProt/UCrea ratio, plasma TBARS, and slightly reduced SBP in 2K1C hypertensive rats, producing no effect in control animals. In 2K1C rats treated with boldine the levels of α-SMA, Col III, ED-1, and OPN were lower when compared to 2K1C rats. Boldine prevented the increase in ACE-1 and TGF-β in 2K1C rats, suggesting that boldine reduces kidney damage. These results suggest that boldine could potentially be used as a nutraceutic.

## 1. Introduction

Hypertension is one of the most common complications that predispose to other health problems, affecting several organs [1]. Human renovascular disease is considered a form of secondary hypertension, in which the activation of the renin-angiotensin system (RAS) has been observed, as a result of the fall in renal blood flow and perfusion pressure due to renal artery stenosis [2,3]. The kidneys respond to this low pressure in the renal vessels producing hormones that lead to sodium and water retention, which causes a rise in blood pressure [4].

Hypertensive nephropathy begins in the glomerulus with an increase in intraglomerular pressure. Therefore, mesangial cells, epithelial cells, and podocytes in the glomerulus are damaged and activated, producing vasoactive and pro-inflammatory agents. These compounds increase cell damage and promote fibrosis, which causes a reduction in renal blood flow, in the permeability of the filtration barrier and, finally in glomerular filtration [1]. This type of hypertension is increasingly related to the pathogenesis of chronic kidney disease (CKD) [3].

CKD is a life-threatening condition characterized by progressive and irreversible loss of renal function. It is manifested by an advancing decrease in the glomerular filtration rate (GFR), resulting from a rise in damaged nephrons and the failure of the organ’s hormonal functions [5]. CKD has different etiologies. Independent of the cause, however, the morphological characteristics, such as tubular necrosis and glomerular sclerosis, are similar [6].

CKD is a condition whose prevalence has increased worldwide. It is estimated that over 10% of adults in developed countries suffers some degree of kidney damage [7], and it has also been reported that 40% of hypertensive patients with renal damage in its terminal stage have a renal artery stenosis [3]. In this sense, animal models have emerged as important tools for understanding the mechanisms implicated in the pathogenic process, and for the assay of prospective therapies.

The Goldblatt two-kidney, one-clip (2K1C) rat hypertension model is a long-established and widely employed model in the study of renal artery stenosis and renovascular hypertension (RVH) [8]. The 2K1C model, not only provides a reproducible and clinically relevant model of systemic hypertension, but exhibits a prominent divergence of biological responses in the two kidneys, as well. It has been described that the stenotic kidney displays progressive atrophy, whereas the contralateral kidney increases in size [3,9]. This divergent behavior of the two kidneys is of special relevance to CKD, because some nephrons exhibit injury and atrophy, whereas other nephrons are enlarged and hyperfunctioning, which can progress to chronic injury, nephron atrophy, and, ultimately, progressive kidney disease [3].

The RAS is the prototype of a classic systemic endocrine network whose actions in the kidney and adrenal glands regulate blood pressure, intravascular volume and electrolyte balance [10]. We have seen that the RAS, including angiotensin II (AngII), has a close relationship with the 2K1C model, since their levels were elevated in the development and maintenance of hypertension in this model [9].

AngII is a key mediator of CKD. It is now understood that AngII mediates renal fibrosis by stimulating endogenous synthesis of transforming growth factor-β (TGF-β) [11]. TGF-β is a cytokine that acts locally in a paracrine form, stimulating the synthesis of the extracellular matrix (ECM), and inhibits the action of matrix-degrading proteases [10]. It has been found that in the development of renal fibrosis, TGF-β is closely associated with the activation of RAS, as AngII induces the transcription and synthesis of TGF-β in damaged kidney cells. Once expressed, TGF-β induces the transformation of fibroblasts into myofibroblasts (α-smooth muscle actin-positive cells, α-SMA) and stimulates the expression of fibronectin (FN) and collagen type III (Col III). This induces the development of fibrosis in the kidney, causing deterioration of the renal function and increasing renal damage [10,12,13].

The steady increase in AngII levels impairs renal function favoring the development of renovascular hypertension [14]. Moreover, if high levels of AngII are kept for extended periods of time, an inflammatory response, characterized by the infiltration of macrophages (ED-1), tubular overexpression of a macrophage chemotactic and adhesion molecule, such as osteopontin (OPN), and the expression of inflammatory factors, such as cytokines, can be induced. This response is ultimately associated with renal damage induced by hypertension [1,14].

The activation of the RAS is crucial in the development and progression of hypertensive renal damage. It has been observed that AngII stimulates the expression of NADPH oxidase, one of the most important enzymes involved in the production of reactive oxygen species (ROS) in the repairing tissue, and contributing to the development of oxidative stress (OS) in various organs [15]. OS is defined as tissue damage caused by increased generation of oxidant compounds combined with a decrease in antioxidant mechanisms. It has been proposed that OS is involved in several pathological conditions, such as cardiovascular diseases, infections, cancer, diabetes, neurodegenerative disorders, and during kidney damage [16]. It has also been demonstrated that OS can induce inflammation, endothelial dysfunction, tissue damage, and hypertension, all of which have been considered to contribute to hypertensive kidney disease [15].

As it has been demonstrated that the RAS participates in the development of the pathology, angiotensin-receptor antagonists or angiotensin converter enzyme (ACE) inhibitors have been commonly used as therapeutic treatments in renovascular hypertension [4]. In addition, recent work, both in humans and in experimental renovascular hypertension animal models, demonstrate that long-term treatment with antioxidants improves both hypertension and the functional alterations in the kidney and heart [4].

Nutrients found in natural products, have been considered to have the potential therapeutic effect controlling the pathogenesis of chronic diseases caused by oxidative stress [17]. Several studies have demonstrated that phenolic compounds help to maintain physiological homeostasis. Polyphenolic compounds which are commonly found as dietary components and particularly polyphenols are useful in scavenging oxygen and nitrogen reactive species [17,18]. In fact, the free-radical scavenging activity of these molecules has been associated with specific structural elements [18]. They can modulate genes, cell metabolism, stress defense, detoxification, and transporter proteins. Moreover, several studies have proven that these extracts produce good results on human health preventing diseases, such as atherosclerosis, diabetes and even cancer [18]. Polyphenolic compounds can prevent the damaging effects of chronic diseases and can delay degenerative ageing. Their mechanisms of action is complex, and involves their structure, their redox status, and the interactions with other agents [17,18].

An important participation of oxidative events mediated by free radicals in the initiation and/or progression of kidney disease has led to the search for new antioxidant molecules [19]. Therefore, the potential to prevent or delay the adverse effects associated with excessive production of ROS by using previously unexplored plant products has proven to be an attractive target for investigation [19].

*Peumus boldus* (i.e., Boldo) is a tree native to central and southern Chile. Dried Boldo leaves have been reported to contain alkaloids in the 0.25–0.54% or 0.4–0.5% range, of which approximately 12–19% is boldine. Boldo bark is an unusually rich source of alkaloids, of which boldine represents about 75% [20]. A large set of pharmacological activities has been attributed to boldine, such as cell protection, as well as anti-inflammatory and antipyretic effects [19,20]. In addition, boldine could scavenge highly-reactive free radicals. The latter has made it possible to postulate that boldine is a nutraceutical product with the potential to be a cellular protector against oxidative damage [19]. Based on the above-mentioned evidence, this study assessed the effect of boldine on the progression of kidney disease in the renal hypertensive rat model 2K1C and identifying mediators in the RAS such as TGF-β, which could be modulated by this alkaloid.

## 2. Results

### 2.1. Boldine Improves Kidney Function and Decreases OS without Decreasing Systolic Blood Pressure (SBP)

Tubulointerstitial and glomerular fibrosis develops in patients and animals with hypertension causing the deterioration of renal function, which ultimately leads to organ damage [15]. Quantification of proteinuria is a key element in the diagnosis and treatment of chronic kidney disease. It is also used to monitor the progression of kidney disease or the response to treatment [16]. For this purpose, a 24 h collection of urine (or around this time) is considered necessary to have an accurate measurement of urinary proteinuria [16,21]. In contrast, a single sample of urine in which creatinine and protein are measured, can also give information about the condition of the kidney. Therefore, we complemented the study of kidney damage by measuring urinary creatinine, allowing us to calculate the U Prot/U Crea ratio, a parameter also used to measure renal function [21].

It has also been shown in several hypertensive models that renal OS may contribute to many of the changes that are associated with the development of hypertensive renal disease, such as inflammation, endothelial dysfunction, tissue damage and hypertension [15]. With these considerations in mind, we evaluated kidney function and oxidative stress in hypertensive 2K1C rats and determined whether the antioxidant properties of boldine, could improve kidney function.

**Figure 1:**
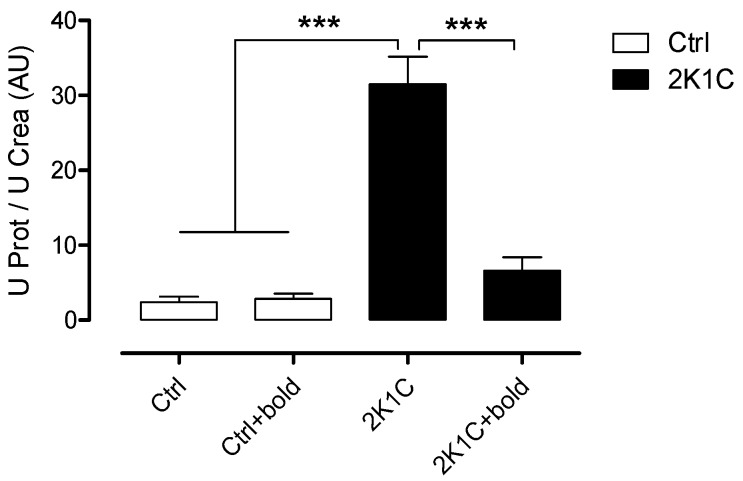
The renal function is improved in 2K1C hypertensive rats treated with boldine. Protein and creatinine were measured in urine samples to assess renal function. Bars represent the mean ± SE. The differences were evaluated by analysis of variance followed by the Tukey post-hoc test. *** *p* < 0.001 vs. 2K1C (*n* = 5 per group).

The U Prot/U Crea ratio (Figure 1) increased significantly in 2K1C hypertensive rats (2K1C; 31.5 ± 8.2 AU), compared to control rats (Ctrl; 2.4 ± 1.6 AU; Ctrl + bold; 2.8 ± 1.6 AU), but these levels were significantly lower in 2K1C hypertensive rats that were treated with boldine (2K1C; 6.6 ± 4.0 AU). In Table 1, which shows the individual values of each parameter measured to assess the renal function of these rats, it can be observed that the major effect of boldine in 2K1C hypertensive rats is the reduction of proteinuria and the fractional excretion of Na^+^ and K^+^ and increase of creatinine clearance to similar values as those present in the control rats.

**Figure 2:**
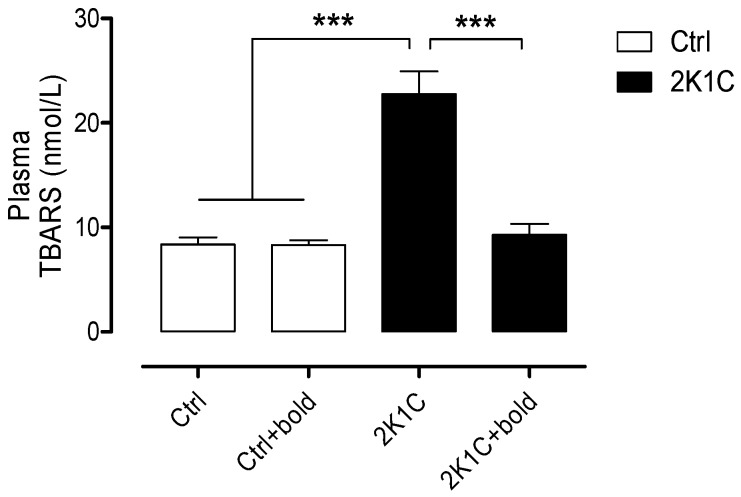
Boldine treatment normalizes plasma levels of TBARS in 2K1C hypertensive rats. Plasma from the four groups was used to measure reactive species of thiobarbituric acid (TBARS) as a measurement of ROS. Bars represent the mean ± SE. The differences were evaluated by analysis of variance followed by the Tukey post-hoc test. *** *p* < 0.001 vs. 2K1C (*n* = 5/all groups).

OS was evaluated by measuring TBARS, where the levels (Figure 2) in plasma of 2K1C hypertensive rats (2K1C; 22.8 ± 4.9 nmol/L) were significantly increased when compared with the control rats (Ctrl; 8.4 ± 1.5 nmol/L; Ctrl + bold 8.3 ± 1.0 nmol/L), but these levels were significantly lower in the plasma of 2K1C hypertensive rats treated with boldine (2K1C + bold; 9.3 ± 2.3 nmol/L).

**Figure 3:**
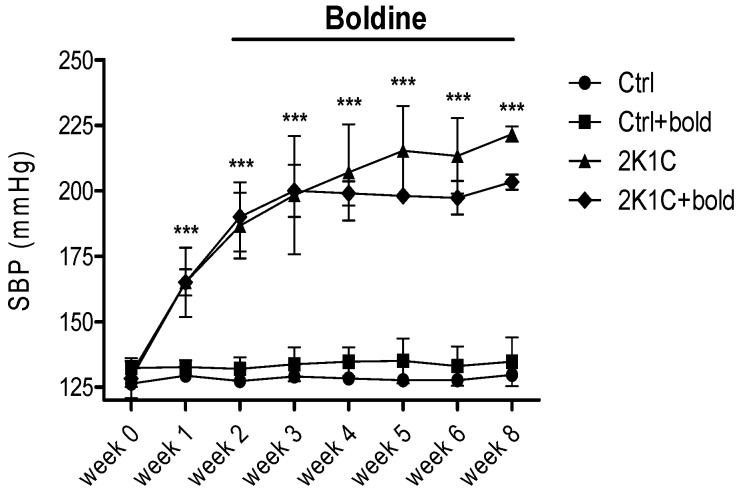
Boldine does not reduce SBP in 2K1C hypertensive rats. Once 2K1C animals became hypertensive (pressure ≥ 180 mmHg; week 2), a randomly selected group of animals (*n* = 5) received the oral treatment with boldine for six weeks (from week 0 to week 6). Pressure was measured weekly until the day of sacrifice. Bars represent the mean ± SE. The differences were evaluated by analysis of variance followed by the Tukey post-hoc test. *** *p* < 0.001 vs. Ctrl (*n* = 3/all groups).

SBP was measured once a week to determine the effect of boldine in this parameter in 2K1C hypertensive rats. As shown (Figure 3) in 2K1C hypertensive rats and in the boldine-treated group (2K1C + bold) SBP was higher (≥200 mmHg) compared with control rats (average values between 130 and 140 mmHg). Boldine treatment was begun on the third week. From the third week until the sixth week of the study, 2K1C hypertensive rats treated with boldine displayed a slightly lower SBP than 2K1C hypertensive rats, which was not significantly different from the 2K1C hypertensive rats. This allows us to suggest that boldine improves renal function, reduces oxidative stress, and has no effect on pressure in 2K1C hypertensive rats.

### 2.2. Boldine Reduces Renal Tissue Damage and Inflammation in Hypertensive 2K1C Rats

We wanted to evaluate the effect of boldine on morphological markers of renal tissue damage such as collagen type III (Col III) and α-smooth muscle actin (α-SMA). The distribution of collagen was assessed by Sirius Red staining quantification from semi-quantitative morphometric analysis.

Evidence from several kidney diseases shows that infiltration of macrophages is closely associated with tubular expression of osteopontin (OPN). OPN is a potent chemoattractant protein that is expressed during renal damage and acts as an adhesion molecule for monocytes and macrophages [22]. For this reason, we studied macrophage infiltration (ED-1) and tubular expression of OPN in 2K1C hypertensive rats.

**Figure 4:**
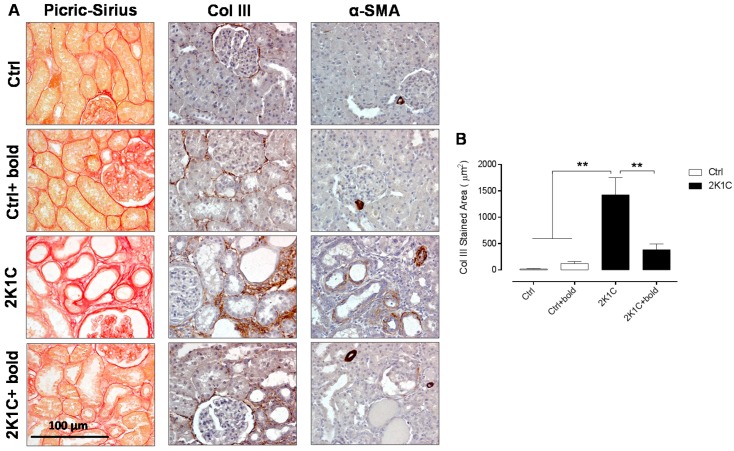
Boldine reduces renal tissue damage in 2K1C hypertensive rats. (**A**) Sirius Red staining, α-SMA, and Col III immunostaining were performed in renal samples six weeks after 2K1C rats were made with hypertension; (**B**) Morphometric analysis of Col III staining in interstitial renal tissue. Representative pictures of at least three different kidneys are shown. Scale bar = 100 μm. Red staining depicts collagen, while brown staining depicts α-SMA or Col III. The differences were evaluated by analysis of variance followed by the Tukey post-hoc test. ** *p* < 0.01 vs. 2K1C (*n* = 5/all groups).

First, the distribution of all collagens was evaluated with Sirius Red staining. We observed an increase in the amounts for this marker in 2K1C hypertensive rats (Figure 4A); however, a reduced collagen distribution was observed in 2K1C rats treated with boldine, like that observed in control rats (Figure 4A). These results were complemented by semi-quantitative morphometric analysis (Table 2).

Immunostaining for α-SMA and Col III was increased in 2K1C hypertensive rats, however, the amounts were lower in 2K1C + bold, like what was observed in control rats, respectively (Figure 4A). These results were also complemented by semi-quantitative morphometric analysis for α-SMA and Col III (Table 2). The morphometric analysis of Col III staining (Figure 4B) increased significantly in 2K1C hypertensive rats (2K1C; 1424.0 ± 327.3 μm^2^), compared to control rats (Ctrl; 18.0 ± 9.5 μm^2^; Ctrl + bold; 121.5 ± 36.6 μm^2^), but these levels were significantly lower in 2K1C hypertensive rats that were treated with boldine (2K1C; 384.3 ± 109.7 μm^2^) (Figure 4B).

**Figure 5:**
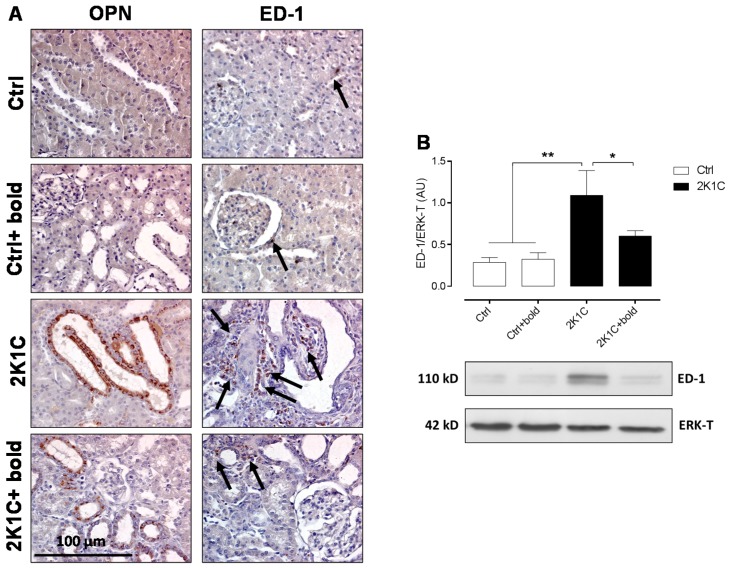
Boldine reduces levels of OPN and ED-1 in 2K1C hypertensives rats. Immunostaining of OPN and ED-1 were performed in renal samples six weeks after 2K1C rats were made hypertensive (**A**); Representative pictures of at least three different kidneys are shown. Scale bar = 100 μm. Brown staining represents the markers OPN and ED-1. For ED-1, the staining is indicated by black arrows. A Western blot for ED-1 is shown (**B**), with pictures of ED-1 bands and T-ERK used as loading control. Bars represent the mean ± SE. The differences were evaluated by analysis of variance followed by the Tukey post-hoc test. * *p* < 0.05 and ** *p* < 0.01 vs. 2K1C (*n* = 5/all groups).

The amounts of macrophages (ED-1) and tubular expression of OPN were increased in 2K1C hypertensive rats (Figure 5A). Nonetheless, lower amounts for these markers were observed in 2K1C hypertensive rats treated with boldine, like control rats and Ctrl + bold rats (Figure 5A). Results obtained by immunohistochemistry were complemented by semi-quantitative morphometric analysis for OPN and ED-1 (Table 3). Western blot for ED-1 (Figure 5B), showed similar results, where protein levels were significantly higher in 2K1C rats (1.1 ± 0.1 AU), and decreased in 2K1C rats treated with boldine (0.6 ± 0.1 AU), to control levels (in AU: Ctrl, 0.3 ± 0.1; Ctrl + bold, 0.3 ± 0.1) (Figure 5B). These results demonstrate that boldine decreases renal injury and inflammation markers, reducing kidney damage in 2K1C hypertensive rats.

### 2.3. TGF-β, Which Has Been Linked to Kidney Damage, Was Reduced by Boldine Treatment

The RAS is a classic endocrine system present in the kidney and acting at the adrenal gland to regulate electrolyte balance, blood pressure, and intravascular volume [10]. Angiotensin II (AngII), an octapeptide produced by renal cells, not only stimulates aldosterone secretion, but induces cellular infiltration, proliferation and migration, thrombosis and reactive oxygen species (ROS) production, as well. In addition, it participates in the induction of the inflammatory response characteristic of nephropathy [1]. It has been found that, in the development of renal fibrosis, TGF-β is closely associated with the activation of RAS, due to AngII stimulation of TGF-β transcription and synthesis in the damaged kidney cells [13]. Therefore, AngII is a key mediator in chronic renal damage participating in the development of fibrosis through stimulation of TGF-β [11]. With these antecedents, we wanted to explore the effects of boldine on RAS mediators, such as angiotensin converting enzyme-1 (ACE-1) and TGF-β, in the 2K1C hypertensive rats.

**Figure 6:**
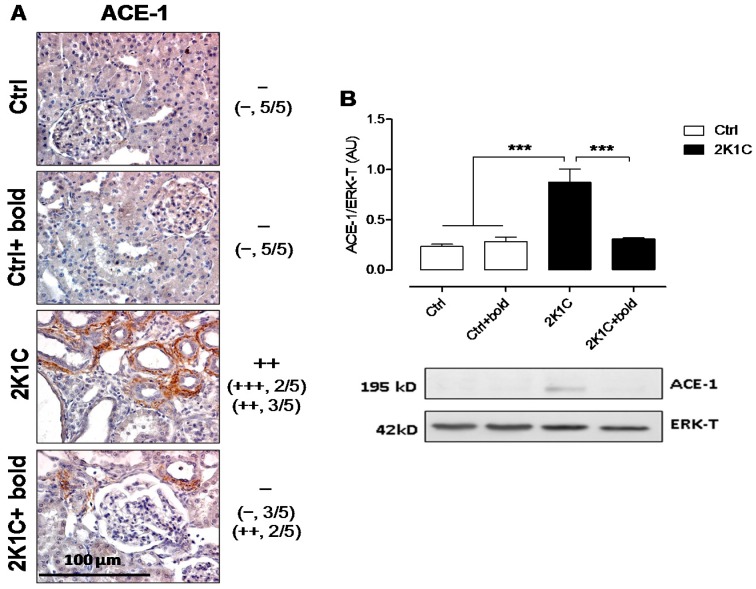
Boldine decreased ACE-1 expression in renal tissue of 2K1C hypertensive rats. Immunostaining for ACE-1 was performed in renal samples six weeks after 2K1C rats were made with hypertension (**A**). Representative pictures of at least five different kidneys are shown. Scale bar = 100 μm. Brown staining represents ACE-1 presence. Morphometric semi-quantitative analysis for ACE-1 was performed on a scale of negative to three crosses (− to +++), defined according to the degree of immunoreactivity observed in the renal tissue sample ([−] = 0–10%, [+] = 10–40%, [++] = 40–70% and [+++] = 70–100%) (**A**); In (**B**) a Western blot for ACE-1 is shown, with pictures of ACE1 bands and T-ERK used as the loading control. Bars represent the mean ± SE. The differences were evaluated by analysis of variance followed by the Tukey post-hoc test. *** *p* < 0.001 vs. 2K1C (*n* = 5/all groups).

We found that, in 2K1C hypertensive rats, ACE-1 distribution was increased, however, the levels were lower in 2K1C + bold, as observed in control rats, respectively (Figure 6A). These results were complemented by semi-quantitative morphometric analysis for ACE-1 (Figure 6A). Western blot for ACE1 (Figure 6B) showed similar results, where protein levels were significantly higher in 2K1C rats (0.9 ± 0.1 AU), and decreased in 2K1C rats treated with boldine (0.3 ± 0.1 AU), to control levels (in AU: Ctrl, 0.2 ± 0.0; Ctrl + bold, 0.3 ± 0.1).

**Figure 7:**
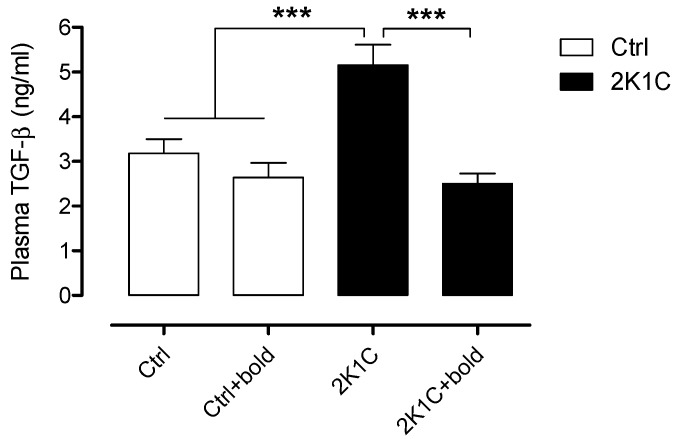
Boldine decreased TGF-β expression in renal tissue of 2K1C hypertensive rats. A TGF-β ELISA assay was performed to determine the TGF-β amounts secreted to plasma under different conditions, six weeks after rats became hypertensive. Bars represent the mean ± SE. The differences were evaluated by analysis of variance followed by the Tukey post-hoc test. *** *p* < 0.001 vs. 2K1C (*n* = 5/all groups).

A TGF-β ELISA assay was performed to determine plasma TGF-β amounts (Figure 7). We observed significantly higher amounts of TGF-β in 2K1C rats (5.2 ± 0.5 ng/mL), however, in 2K1C hypertensive rats treated with boldine, TGF-β amounts (2.5 ± 0.2 ng/mL), were closer to amounts observed in control rats (In ng/mL: Ctrl, 3.2 ± 0.3; Ctrl + bold 2.6 ± 0.3). These results suggest that boldine reduces renal damage by reducing ACE-1 and TGF-β amounts in 2K1C hypertensive rats.

## 3. Discussion

The incidence of chronic kidney disease (CDK) is increasing worldwide and the current available therapies cannot reduce this phenomenon [23]. Current therapies focus on blood pressure control and optimizing the blockade of the renin-angiotensin-aldosterone system [24]. However, these treatments are partially effective in advanced and late stages of kidney disease.

The Goldblatt two-kidney, one-clip (2K1C) rat hypertension model exhibits two facets of this model: atrophy of one kidney and increased growth of the other [3]. These are of special relevance to CKD, wherein a certain subset of nephrons exhibit injury and atrophy, whereas other nephrons display structural enlargement and hyperfunction, since the latter may serve as a precursor to chronic injury, nephron atrophy, and, ultimately, progressive kidney disease [25].

The quantification of proteinuria is a fundamental element in the diagnosis and treatment of renal disease. It is also used to monitor the progress of kidney disease or response to treatment. However, it has important limitations that make this parameter is not used to assess kidney damage in advanced stages. This is complemented by the study of kidney damage by measuring creatinine, designed to measured kidney function [21].

To evaluate the degree of renal damage, which is characteristic of patients with CDK, renal function was assessed by measuring the ratio of U Prot/U Creat (Figure 1), which strongly correlates with protein excretion in 24 h [26,27]. Treating in 2K1C rats with boldine (50 mg/kg/day) resulted in a significant decrease of this ratio. This allows us to suggest that boldine prevents the loss of renal function, maintaining glomerular filtration and preventing kidney damage during CDK.

The pathophysiological basic mechanisms of renal disorders are associated with factors that predispose to oxidative and inflammatory imbalance [28,29]. The phenomena that occur during CKD that can damage the tubule and the glomerulus can lead to the generation of reactive oxygen species (ROS), such as TBARS [30,31,32].

Boldine has been demonstrated to be a potent antioxidant for its ability to scavenge HO· radicals in several experimental models [19]. Moreover, it has been shown that boldine possess significant hepatoprotective potential in various models of toxic liver injury acting as agonist of the Farnesoid X receptor (FXR) and producing sustained mild bile acid (BA)-dependent choleresis by upregulating the bile salts export pump (Bsep) [33,34,35]. With this background, we evaluated ROS levels in 2K1C hypertensive rats by measuring TBARS (thiobarbituric acid reactive species) and observed a decrease in OS. The reduction in TBARS (Figure 2) suggests that boldine is reducing OS, and by doing that it is reducing the amount of species that can react with thiobarbituric acid in the plasma of 2K1C hypertensive rats. These results are consistent with those observed in other studies, where the antioxidants quercetin, resveratrol, bardoxolone methyl, and gamma-aminobutyric acid were used and improved GFR in rats with renovascular hypertension [4,36,37,38].

Sustained high blood pressure is one of the most powerful determinants of renal hypertrophy [4]. In our study, the 2K1C rats treated with boldine showed a weak decrease in SBP as compared to the 2K1C group (Figure 3). This weak reduction was far from normal values. However, this effect was accompanied by decreased lipid peroxidation and improved renal function. This fact suggests that boldine possesses antihypertrophic and renoprotector properties unrelated to its antihypertensive effect, contrary to what was observed in the work of Lau et al., where boldine treatment significantly lowered SBP in spontaneously hypertensive rats (SHR) [39].

Renovascular hypertension is increasingly related to the pathogenesis of CKD [3]. CDK is associated with the development of renal interstitial fibrosis and is characterized by atrophy and/or tubular dilatation and increased interstitial matrix deposition [40]. In addition, in a wide range of renal diseases, macrophage infiltration (ED-1) is closely related to the regulation of tubular expression of osteopontin (OPN). OPN is a potent chemoattractant that is expressed during kidney damage and acts as an adhesion molecule for monocytes and macrophages [12,22]. Additionally, it is believed that the development of interstitial fibrosis is the cause of the irreversibility of renal dysfunction [41,42]. Although the participation of different cells has been postulated, myofibroblasts (which express α-SMA and Col III) are the main cells that participate in renal fibrosis [43]. If this hypothesis is true, we could speculate that boldine, due to its antioxidant properties [19], could improve renal damage and inflammation. For this reason, we evaluated the effect of boldine on the expression of characteristics markers of kidney damage such as α-SMA and Col III, and inflammation markers such as ED-1 (macrophage infiltration) and OPN, respectively. Our results (Figure 4 and Figure 5; Table 2 and Table 3) show that the increase in markers of kidney damage and inflammation were lower in 2K1C rats treated with boldine when compared to untreated 2K1C rats. Accordingly, we can postulate that boldine reduces fibrosis and inflammation in kidney damage in 2K1C hypertensive rats.

The activation of the renin-angiotensin system (RAS) is a crucial factor in the development and progression of organ damage in hypertension, diabetes and CDK [25,28,44]. Accumulating evidence over the past several years suggest that TGF-β plays a pivotal role in the progression of many immune and non-immune-mediated renal diseases [13,42,45]. It is known that TGF-β is involved in the development of fibrosis [13], induces inflammatory response which is characterized by the infiltration of macrophages (ED-1) and over-expression of OPN [12,41], and can induce the expression of the NADPH-OX, one of the key enzymes in the ROS generation, contributing to OS [15]. In addition to TGF-β, AngII also contributes to the progression of renal disease through hemodynamic, as well as nonhemodynamic mechanisms [3,13]. These two apparently diverse factors, which are involved in the deterioration of renal function and structure during CKD, may be joined together by the observation that AII induces TGF-β expression [3,13]. Ozawa et al., in 2007, observed that in a model of Sprague Dawley rats stimulated with AngII for six days, and then three or six days without the stimulus, respectively, presented interstitial fibrosis, glomerular hypertrophy, infiltration of macrophages, an increase in the amount of mRNA of the chemoattractant protein MCP-1, and also in mRNA quantity of transforming growth factor-β (TGF-β) [1]. It is also known that TGF-β stimulates the synthesis of extracellular matrix (ECM) and inhibits the action of proteases that degrade the matrix [10,11], influencing the development of fibrosis and inflammation in the kidney, causing deterioration of function and increasing renal damage [10,12,13]. In addition, TGF-β has been shown to have a close relationship with RAS in the development of hypertension and kidney damage in the 2K1C Goldblatt hypertensive model [3]. With this background, we wanted to study what happened to the mediators of RAS, such as ACE-1 (Figure 6) and TGF-β (Figure 7). According to the results the increase in ACE-1 and TGF-β were lower in 2K1C rats treated with boldine than that observed in untreated 2K1C rats.

In the present study, despite normalizing renal function and OS, reducing fibrosis, inflammation, and RAS mediators; boldine treatment was not able to normalize the elevated SBP in 2K1C rats. Currently we have been able to identify the exact mechanism of this discrepancy; however, it has been described that several OS-independent factors, such as, alterations in the sympathetic nervous system, can additionally participate in the development of hypertension in 2K1C rats [39].

## 4. Conclusions

**Figure 8:**
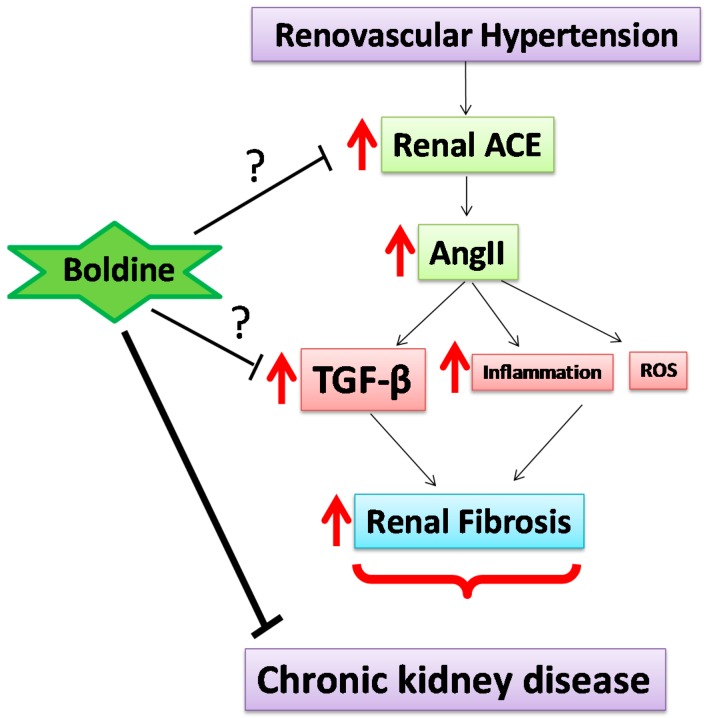
Mechanism in which the 2K1C hypertensive rat model leads to the development of renal damage and boldine would cause the reduction of kidney damage. RAS is a key mediator in chronic kidney damage as it participates in the development of fibrosis by stimulating the synthesis of TGF-β. In addition, high levels of Ang II maintained for long periods can induce the inflammatory response and generation of ROS, contributing to the onset of oxidative stress (red arrows). Therefore, Ang II is a key mediator in chronic kidney disease as it participates in the development of fibrosis by stimulating the synthesis of TGF-β (red arrow). Boldine could prevent the increase in ACE-1, causing a decrease in all downstream mediators, including TGF-β and mediators downstream of this marker; and could even prevent the same increase of TGF-β (question mark), suggesting that boldine reduces kidney damage affecting the RAS in the kidney of 2K1C rats.

In conclusion, these results suggest that boldine prevents the increase in ACE-1, causing a decrease in all downstream mediators, including TGF-β and mediators downstream of this marker, suggesting that boldine reduces kidney damage affecting the RAS in the kidney of 2K1C rats (Figure 8). We postulate that due to its properties, boldine has a high potential to be used as a nutraceutical compound in the treatment of renal problems.

## 5. Methods

### 5.1. Antibodies and Chemicals

The following primary antibodies were used: monoclonal antibodies against macrophages (clone ED-1) were obtained from AbD Serotec (Kidlington, UK); antibodies against α-smooth muscle actin (α-SMA) were obtained from Sigma-Aldrich (St. Louis, MO, USA); antibodies against collagen type III (Col III) and angiotensin converting enzyme-1 (ACE-1) were from Santa Cruz Biotechnology (Santa Cruz, CA, USA); and osteopontin (OPN) antibody (MPIIIB101) was provided by the laboratory of Dr. Carlos Vío (developed by DSHB, Iowa City, IA, USA). Secondary antibodies and the corresponding PAP complexes were purchased from ICN Pharmaceuticals-Cappel (Costa Mesa, CA, USA). Triton X-100, 3.3′-diaminobenzidine, λ-carrageenan, Tris-HCl, hydrogen peroxide, phosphate salts, and other chemicals were purchased from Sigma-Aldrich (St. Louis, MO, USA).

### 5.2. Animals and Experimental Protocol

Adult male Sprague–Dawley rats (100–120 g) were maintained under a 12 h light/12 h dark cycle, with food (20.5% protein, 5% fiber, 4% fat; Champion, Santiago, Chile) and water ad libitum at the university animal care facilities. All procedures were in accordance with institutional and international standards for the humane care and use of laboratory animals (Animal Welfare Assurance Publication A5427-01, Office for Protection from Research Risks, Division of Animal Welfare, NIH (National Institutes of Health), Bethesda, MD, USA), as described previously [46]. The development of this manuscript is part of the objectives of the project “Effect of two phytopharmaceuticals with antioxidant properties on the progression of renal disease” (VRI N2/2011), which has the approval of the Bioethics Committee of the Faculty of Biological Sciences of the Pontificia Universidad Católica de Chile. Rats were randomly divided into four groups: control (Ctrl, *n* = 5), control + boldine (Ctrl + bold, *n* = 5), 2K1C Goldblatt (2K1C, *n* = 5), and 2K1C Goldblatt + boldine (2K1C + bold, *n* = 5). We used the 2K1C hypertensive model previously described by the group of Guan et al., 1992. Rats were anaesthetized with ketamine/xylazine (10:1 mg/kg of body weight, intraperitoneal); then, a retroabdominal incision in the left flank was performed, the left kidney was exposed. Afterwards, the left renal artery of 2K1C Goldblatt-groups animals was occluded by a silver clip (0.2 mm internal diameter of the vessel), while the right kidney was not disturbed. Control-group rats were sham-operated by an incision made in the flank [9]. After recovery, rats were maintained with food and water ad libitum. Before starting treatment with boldine, Hypertension was confirmed by measuring systolic blood pressure (SBP) weekly with a non-invasive plethysmography (NIBP machine, IITC Inc., Woodland Hills, CA, USA) until SBP was ≥180 mmHg. Once 2K1C animals became hypertensive (pressure ≥ 180 mmHg), a group of them (*n* = 5) was selected at random to receive the oral treatment with boldine. Boldine (50 mg/Kg/day), or its vehicle (water), were given by gavage over six weeks. Administration was stopped one day before the end of the experiments to study boldine long-term effects without the involvement of its acute administration effects. The last day of the experimental period animals were placed in metabolic cages for 16 h to collect urine in a container built into the cage. The next morning, rats were anesthetized with ketamine/xylazine (10:1 mg/kg of body weight, ip). Blood samples were obtained from the abdominal aorta, centrifuged, and plasma was frozen for further analysis. Urine was measured and aliquoted. Kidneys were processed for immunohistochemistry and Western blotting. Animals were sacrificed by exsanguination under anesthesia.

### 5.3. Blood Pressure Measurements

SBP was determined once a week, in the morning, in conscious pre-warmed restrained rats by a non-invasive plethysmography (NIBP machine, IITC Inc., Woodland Hills, CA, USA). At least four determinations were made in every session and the mean of the four values was taken as the SBP value.

### 5.4. Renal Function Measurements

Plasma and urinary creatinine levels were measured with the Jaffé alkaline picrate assay (VALTEK Diagnostica, Santiago, Chile). Urinary protein concentration was determined by Bradford’s method (Bio-Rad protein assay, Kidlington, UK) [47]. Creatinine clearance over 24 h was calculated according to the standard formula *C* = (*U* × Ṽ/*P*, where *C* is the creatinine clearance, *U* is the creatinine urinary concentration, Ṽ is the urine flow rate per minute, and *P* is the creatinine plasmatic concentration [46].

### 5.5. Thiobarbituric Acid Reactive Substances (TBARS) Measurement

TBARS were quantified by using a modified version of the method published by Ramanathan and collaborators. Supernatant from cells in culture, was mixed with acetic acid (20% *v*/*v*), SDS (8% *w*/*v*), and thiobarbituric acid (0.8% TBA *w*/*v*), and heated at 90 °C for 60 min. Afterwards, the mixture was centrifugated to remove precipitated material, and absorbance was measured at 532 nm. TBARS were estimated with a calibration curve of malondialdehyde tetrabutylammonium salt (MDA) used as standard. MDA was obtained from Sigma-Aldrich (#36357, St. Louis, MO, USA).

### 5.6. Histological Damage Assessment

Tissue damage was evaluated by a semi-quantitative morphometric analysis that was performed according to the degree of immunoreactivity observed in the kidney tissue sample for each of the antibodies used (OPN, ED-1, α-SMA, Col III). This was done from a score on a scale from negative to positive three (− to +++), defined according to the degree of immunoreactivity observed in the kidney tissue sample ([−] = 0–10%, [+] = 10–40%, [++] = 40–70% y [+++] = 70–100% of the area observed). To assess the degree of fibrosis, staining of collagen fibrils by Sirius red F3BA was carried out as previously described [48].

### 5.7. Tissue Processing and Immunohistochemical Analysis

Renal slices (3 mm-thick), including the cortex, medulla, and papilla, from different groups, were fixed for 24 h by immersion in Bouin’s solution at room temperature. The tissue was then dehydrated, embedded in Paraplast plus (Monoject Scientific, St. Louis, MO, USA), serially sectioned at 5-mm thickness with a rotatory microtome, mounted on glass slides, and stored until immunostaining. Immunolocalization studies were performed using an indirect immunoperoxidase technique [49]. Tissue sections were quickly dewaxed, rehydrated, rinsed in immune solution (IS) (0.11 M Na_2_HPO_4_, KH_2_PO_4_ 0.04 M, 1 M NaCl, 0.32 M Tris-HCl, and 0.03 M sodium azide) pH 7.6, and incubated overnight at 22 °C with the primary antibody (1:100). Afterward, sections were washed three times with IS for 5 min each, followed by 30 min incubation at 22 °C with the corresponding secondary antibody (1:20) and with the peroxidase-antiperoxidase (PAP) complex (1:150). Immunoreactive sites were revealed using 0.1% (*w*/*v*) 3.3′-diaminobenzidine and 0.03% (*v*/*v*) hydrogen peroxide solution. The antisera and PAP complex was diluted in IS containing 0.25% (*v*/*v*) Triton X-100 and 0.7% (*w*/*v*) λ-carrageenan. The sections were rinsed with IS buffer between incubations, counterstained with hematoxylin, dehydrated and cleared with xylene, and coverslipped. Sections were counterstained with hematoxylin and images acquired using a Nikon Eclipse E600 microscope and Nikon DXM1200 digital camera were analyzed using conventional light microscopy and Nomarski differential interference contrast (DIC) microscopy as previously described [50]. The Col III-stained area in each image was quantified utilizing computer-assisted image-analysis software (Simple PCI, Hamamatsu, Japan). Total immunostained cells were averaged and expressed as the mean absolute values per square micron, as previously described [50].

### 5.8. Western Blot

Renal tissues were homogenized with an Ultra-Turrax homogenizer in buffer containing Tris-HCl 100 mM pH 7.4, EDTA 5 mM, SDS 1%, PMSF 1 μM and the protease inhibitor cocktail (Pierce, Rockford, IL, USA). Protein concentrations were determined by using a detergent-compatible Bio-Rad protein assay kit (Bio-Rad, Richmond, CA, USA). Fifty micrograms (50 μg) of renal proteins were mixed with an equal volume of sodium dodecyl sulfate (SDS)-polyacrylamide gel electrophoresis sample buffer (100 mM Tris-HCl, pH 6.8, 200 mM dithiothreitol, 4% SDS, 0.2% bromophenol blue, 20% glycerol) and were boiled for 3 min. The proteins in the renal samples were separated on 10% SDS-polyacrylamide gels and transferred to either polyvinylidene difluoride or nitrocellulose membranes. Membranes were blocked with blocking solution (5% nonfat dry milk in Tris-buffered saline-Tween) for 30 min at room temperature. Membranes were blotted overnight at 4 °C with monoclonal anti-ACE1 (1:500) and monoclonal anti-ED-1 (1:1000), then were stripped and reblotted with a polyclonal anti-ERK-1/2 (1:2000, Santa Cruz Biotechnology) antibody used as loading control. Immunoreactive bands were visualized by a chemiluminescent method (Western Lightning, Thermo Scientific, Pierce, Rockford, IL, USA) and Kodak X-LS film. The bands detected were digitized and subjected to densitometry analysis using the software Image J (Version 1.50i, NIH, Washington, DC, USA).

### 5.9. Enzyme-Linked Immunosorbent Assay

TGF-β ELISA assays was performed to determine the amount of TGF-β levels secreted in plasma under different conditions, following the manufacturer’s protocol (TGF-β EIA kit, Enzo Life Science, Farmingdale, NY, USA). Results were presented in ng/mL.

### 5.10. Statistical Analysis.

Results were evaluated by ANOVA, and the Tukey’s post-test was used to evaluate the difference between two groups. Results are expressed as the average of values from each independent experiment ± SE and considered significantly different if *p* < 0.05. Analyses were performed with GraphPad Prism 5 software for Windows (1992–2007, GraphPad Software, 12 March 2007, La Jolla, CA, USA).

## Figures and Tables

**Table 1:** Values for proteinuria, creatininuria, creatininemia, creatinine clearance, and fractional excretion (FE) for Na^+^ and K^+^ in the four groups. Boldine was administered by gavage at doses of 50 mg/kg during six weeks after hypertension was established in 2K1C rats (pressure ≥ 180 mmHg). Samples of urine and plasma were obtained. Protein and creatinine were measured to assess renal function; the fractional excretion of sodium and potassium (FENa^+^, FEK^+^, [%]) was also determined. Data are expressed as mean ± SE. The differences were evaluated by analysis of variance followed by the Tukey post-hoc test. * *p* < 0.05, ** *p* < 0.01, *** *p* < 0.001 vs. 2K1C (*n* = 5/all groups).

Groups	Weight (gr)	Proteinuria (mg/day)	Urine Creatinine (mg/day)	Plasma Creatinine (mg/mL)	Creatinine clearence (mL/min)	FE Na^+^ (%)	FE K^+^ (%)
**Ctrl (*n* = 5)**	410 ± 10.5	11.6 ± 2.4 ***	6.4 ± 1.8	0.3 ± 0.0 ***	0.4 ± 0.1 *	0.1 ± 0.0 **	29.6 ± 6.2 **
**Ctrl + bold (*n* = 5)**	412 ± 20.3	15.9 ± 6.0 ***	6.6 ± 1.0	0.2 ± 0.0 ***	0.4 ± 0.1 *	0.1 ± 0.0 **	21.6 ± 1.1 **
**2K1C (*n* = 5)**	383 ± 8.7	132.2 ± 11.7	4.3 ± 0.4	0.8 ± 0.1	0.1 ± 0.0	0.6 ± 0.1	101.6 ± 23.1
**2K1C + bold (*n* = 5)**	390 ± 13.4	26.4 ± 5.8 ***	4.4 ± 0.3	0.3 ± 0.1 ***	0.3 ± 0.1	0.2 ± 0.1 *	30.6 ± 6.0 **

**Table 2:** Immunoreactivity score for Picric-Sirius, α-SMA, and Col III from tissue samples from rats of the four experimental groups. The semi-quantitative morphometric analysis for Picric-Sirius, α-SMA, and Col III was performed on a scale of negative to three crosses (− to +++), defined according to the degree of immunoreactivity observed in the renal tissue sample ([−] = 0–10%, [+] = 10–40%, [++] = 40–70% and [+++] = 70–100%).

	Score		
Groups	Picric-Sirius	Col III	α-SMA
**Ctrl (*n* = 5)**	− (−, 5/5)	− (−, 4/5; +, 1/5)	− (−, 5/5)
**Ctrl + bold (*n* = 5)**	− (−, 4/5; +, 1/5)	− (−, 4/5; +, 1/5)	− (−, 5/5)
**2K1C (*n* = 5)**	+++ (+++, 4/5; ++, 1/5)	++ (+++, 2/5; ++, 2/5; +, 1/5)	+ (+, 4/5; −, 1/35)
**2K1C + bold (*n* = 5)**	+ (+, 5/5)	− (−, 3/5; +, 2/5)	− (−, 5/5)

**Table 3:** Immunoreactivity score for OPN and ED-1 from the tissue samples from rats of the four experimental groups. The semi-quantitative morphometric analysis for OPN and ED-1 was performed on a scale of negative to three crosses (− to +++), defined according to the degree of immunoreactivity observed in the renal tissue sample ([−] = 0–10%, [+] = 10–40%, [++] = 40–70% and [+++] = 70–100%).

	Score	
Groups	OPN	ED-1
**Ctrl (*n* = 5)**	− (−, 5/5)	− (−, 5/5)
**Ctrl + bold (*n* = 5)**	− (−, 3/5; +, 2/5)	− (−, 4/5; +, 1/5)
**2K1C (*n* = 5)**	+++ (+++, 3/5; ++, 2/5)	+++ (+++, 3/5; ++, 2/5)
**2K1C + bold (*n* = 5)**	+ (+, 4/5; ++, 1/5)	+ (−, 2/5; +, 3/5)

## Abbreviations

Renin-Angiotensin System	RAS
Chronic kidney disease	CDK
Glomerular filtration rate	GFR
Goldblatt two-kidney one clip	2K1C
Renovascular hypertension	RVH
transforming growth factor-β	TGF-β
Extracellular matrix	ECM
Alpha-smooth muscle actin	α-SMA
Fibronectin	FN
Collagen type III	Col III
infiltration of macrophages	ED-1
Osteopontin	OPN
Reactive oxygen species	ROS
Oxidative stress	OS
Angiotensin converter enzyme	ACE
Systolic blood pressure	SBP
Enzyme-Linked Immunosorbent Assay	ELISA
